# Ancestral genetic components are consistently associated with the complex trait landscape in European biobanks

**DOI:** 10.1038/s41431-024-01678-9

**Published:** 2024-08-10

**Authors:** Vasili Pankratov, Massimo Mezzavilla, Serena Aneli, Ivan A. Kuznetsov, Daniela Fusco, James F. Wilson, Mait Metspalu, Paolo Provero, Luca Pagani, Davide Marnetto

**Affiliations:** 1 Center for Genomics, Evolution and Medicine, Institute of Genomics, University of Tartu, 51010 Tartu, Estonia; 2 Department of Biology, University of Padua, Padua, Italy; 3 Department of Public Health Sciences and Pediatrics, University of Turin, 10126 Turin, Italy; 4 Department of Neurosciences, University of Turin, 10126 Turin, Italy; 5 Centre for Global Health Research, Usher Institute, University of Edinburgh, Teviot Place, Edinburgh, EH8 9AG Scotland; 6 MRC Human Genetics Unit, Institute of Genetics and Cancer, University of Edinburgh, Western General Hospital, Crewe Road, Edinburgh, EH4 2XU Scotland; 7 Centre for Genomic and Experimental Medicine, Institute of Genetics and Cancer, University of Edinburgh, Western General Hospital, Crewe Road, Edinburgh, EH4 2XU Scotland; 8 Institute of Genomics, University of Tartu, 51010 Tartu, Estonia; 9 Center for Omics Sciences, IRCCS San Raffaele Scientific Institute, 20132 Milan, Italy

**Keywords:** Population genetics, Quantitative trait

## Abstract

The genetic structure in Europe was mostly shaped by admixture between the Western Hunter-Gatherers, Early European Farmers and Steppe Bronze Age ancestral components. Such structure is regarded as a confounder in GWAS and follow-up studies, and gold-standard methods exist to correct for it. However, it is still poorly understood to which extent these ancestral components contribute to complex trait variation in present-day Europe. In this work we harness the UK Biobank to address this question. By extensive demographic simulations, exploiting data on siblings and incorporating previous results we obtained from the Estonian Biobank, we carefully evaluate the significance and scope of our findings. Heart rate, platelet count, bone mineral density and many other traits show stratification similar to height and pigmentation traits, likely targets of selection and divergence across ancestral groups. We show that the reported ancestry-trait associations are not driven by environmental confounders by confirming our results when using between-sibling differences in ancestry. The consistency of our results across biobanks further supports this and indicates that these genetic predispositions that derive from post-Neolithic admixture events act as a source of variability and as potential confounders in Europe as a whole.

## Introduction

In order to uncover the genetic basis of complex traits in Genome Wide Association Studies (GWAS) [1], a large amount of data has been collected in nation-wide population-based Biobanks [1–5]. Despite including predominantly individuals of European ancestry, which poses well-recognized limitations [1, 6–8], these cohorts still contain stratification such as socio-economic disparities, geographic factors and, most importantly, inherent genetic structure, which might result in non-causal associations. A plethora of methods [9–11] have been developed to correct for these unwanted sources of variance that might bias GWAS discovery. Indeed even the finer cases of population structure present in national Biobanks [12–14] have been demonstrated to affect GWAS [14, 15] and, if not carefully addressed, hamper analyses following up on these results, such as polygenic risk scoring [12, 16–19] and polygenic selection testing [20, 21].

Millennia of demographic expansions, migrations, and localized genetic isolation have indeed shaped a far from homogeneous genetic makeup for contemporary Europeans. Besides recent demographic history, predominantly impacting on rare variation structure, a series of admixture events from 8000 BCE to 1000 BCE have been foundational to the European genetic landscape. During that period, Early European Farmers (EEF) and Steppe Bronze Age (SBA) genetic ancestries gradually spread into and across Europe blending with the local Western Hunter-Gatherers (WHG) substratum [22–24], bringing together genetic components that had evolved separately for up to 20,000 years [25]. Divergent phenotypes in these source populations have been previously described for a few traits using polygenic scoring of ancient samples [26–28], and very recently of ancestral segments from modern samples [29], or looking at specific trait-informative Single Nucleotide Polymorphisms (SNPs) [30–32].

These ancestral components can explain a large part of the genetic gradients across Europe [23] and, as a result, are indirectly adjusted for in gold-standard GWAS procedures. In this work, we aim to identify which complex trait variations across the largest European biobank can be explained by the stratification of these ancestral components. Importantly, although genetic predispositions conferred by these components might be indirect and involve pleiotropy and/or complex interactions with the environment, we devote particular care to avoid apparent associations caused by geographic and socio-economic stratifications co-occurring with the ancestral components.

We previously attempted to quantify their differential contribution to the contemporary landscape of complex traits in 35,000 individuals from the Estonian Biobank (EstBB) [33]. Our findings provided the first picture to describe the association of ancestral populations to present day traits; however, it remained unclear whether our conclusions could be applied to the broader European population. In addition, we could not decisively address the differences in the resulting signal when running the analysis over local candidate regions or over the whole genome. Finally, the study lacked a meticulous simulation testing the limits of the adopted statistic in answering our question.

Here we overcome these limitations by analyzing a total of 53 complex traits in 50,000 UK Biobank [2] (UKBB) donors taken as representative of the Western European metapopulation. We validate the ancestry-trait associations found as European-wide signals, find new ones, and explore the strengths and limitations of our approach with thorough simulations using SLiM [34].

## Subjects and methods

### Framework overview

Our approach is based on *covA*, a measure of relative genetic similarity of an individual from a contemporary population to the distinct ancestries (e.g. WHG; EEF; SBA) that contribute to the genetic makeup of that population. This metric was introduced in our previous work [33] for the analysis of complex traits in EstBB and is ultimately a covariance between allele dosage in a contemporary individual and a given ancestral population, with respect to the contemporary and ancient average frequencies. We regress each complex trait *t* in the present-day dataset on the *covA* for each ancestry *p* so that for each individual *i*:

$${t}_{i}={\beta }_{0}+{\beta }_{{{\mathrm{cov}}}A(p)}{{{\cdot }}}{{\mathrm{cov}}}A(i,p)+{\varepsilon }_{i}$$

where the slope ${\beta }_{{{\mathrm{cov}}}A(p)}$ quantifies the association between each ancestry *p* with the trait *t* and ${\varepsilon }_{i}$ represents the error; covariates can potentially be added (see **Models tested and covariates**).

In order to reduce the influence of genomic confounding factors, we compute *covA* restricting to variants belonging to 20 kb genomic regions around SNPs that have been previously associated with the trait of interest through GWAS, i.e. Trait-Associated Genomic Regions (TAGR, see **Traits and candidate regions**). Note that GWAS-derived summary statistics are used only to identify TAGRs: using these summaries as weights in follow-up analyses, especially when summed across the whole genome, has been shown to produce results difficult to interpret in populations that are even subtly genetically differentiated from the ones where the GWAS was run [12, 21, 35, 36].

### Simulations

Below is a brief description of the simulations. See Supplementary Text for further information.

To explore the behavior of *covA* under different heritability, polygenicity, stabilizing selection and differences in trait optima between ancestral populations we performed hybrid simulations using a combination of SLiM [34] and msprime [37]. We simulated 1000 unlinked genomic intervals of 20 kb each (portraying potential TAGRs) with a uniform recombination rate of 1e-8 and a uniform mutation rate of 1.25e-8 [38] under a demographic model relevant for the British population (Fig. S1) with each such interval containing one trait-affecting SNP. In each generation we calculated the genetic value (GV) for each individual based on the genotypes at these SNPs, converted this GV to the trait value by adding environmental noise and then mapped it to fitness using an approach similar to the one used by Yair and Coop, 2022 [39]. All in all, we ran 2370 simulations representing 135 simulation scenarios.

Each simulation run resulted in genotypes and trait values for 10 K contemporary individuals (around 7 K after filtering for relatives) and 100 individuals from each of the three reference populations (WHG, EEF, SBA; no relatives filtering applied). The causal SNPs were ascertained based on the fraction of heritability of the trait they explain to mimic SNP discovery in GWAS, resulting in between 351 and 565 TAGR used to calculate *covA* (Table S1). Finally, the simulated traits and *covAs* were plugged in the regression above without any other covariate.

### Sample selection and definition of ancestral groups

UKBB [2] donors were selected among those (a) identified as British of West-European descent (code 1) according to Data-Field 22006 (which includes self-identification and genetic grouping), (b) selected for the UKBB Principal Component Analysis (Data-Field 22020), which excludes extreme heterozygosity, missingness and up to third-degree relatives; and (c) having at most one missing trait among those analyzed. We then extracted a subset of 50,000 samples equally divided between females and males for the analyses.

To validate our results using differences across siblings, we identified 17,319 sibships (35,585 samples) among the UKBB donors identified as British of West-European descent as above, keeping pairs with kinship coefficient between 0.177 and 0.354 and identified as full siblings by KING v2.2.7 based on IBD2 sharing. In case a donor was involved in more than one sibling pair, we required all donors within such a sibship to be identified as full siblings, otherwise the whole sibship was removed.

Ancient samples were extracted from the Allen Ancient DNA Resource [40] v52.2 (AADR) following the approach described in Marnetto et al. [33]. After starting from a manually curated core set for each ancestral group, we expanded these sets to other AADR samples according to distance in a 4-dimensional space defined by dating and first 3 Principal Components (PC). PCs were determined on a set of modern Eurasian and North African individuals west of Iran (included), where the ancient samples were projected; distance cutoff was defined by multi-dimensional ellipses with diameters equal to 3 core set SDs. See Table S2 for a full list of ancestral group classifications, coordinates, dates and PCs.

We used phased, imputed genotype data for the UKBB set and intersected it with the ancient set, obtaining 1,087,822 genotyped variants in the merged dataset.

### Traits and candidate regions

Traits were selected to span different domains and favor large effective sample sizes, see Table S3 for a complete list accompanied with the original UKBB Field ID. When multiple data points existed for an individual, the earliest one was considered. For each continuous trait, individuals with values more distant than 4 IQRs from the upper or lower quartile were discarded as outliers, then traits were standardized. Some traits were computed (waist/hip ratio, pulse pressure, caffeine, etc.), log-transformed (body mass index (BMI), creatinine,…) or BMI-adjusted (waist and hip circumference, blood pressure, etc.), see Table S3.

Trait-associated genomic regions (TAGRs) were defined starting from GWAS Catalog [41] data, downloaded on 09/05/2023. This resource collects small-scale variant hits which, depending on the original study, either are genome-wide significant (*p*-value < 0.5⋅10^−8^) or genome-wide suggestive (*p*-value < 10^−5^). We selected hits by matching a pattern to the trait definition given by the original study or by selecting a mapped Experimental Factor Ontology term, according to Table S4, then defined 20 kb windows centered on the selected hits and merged them, obtaining a set of TAGRs for each trait.

### Partitioned heritability

In order to assess TAGRs contribution to heritability of traits, a Stratified LD Score Regression (sLDSC) was conducted on UKBB GWAS summary statistics estimated by the Neale Lab (http://www.nealelab.is/uk-biobank/) for 50 complex traits. We used 503 samples of European ancestry from the 1000 Genomes Project [42] to compute LD Scores; HapMap Project Phase 3 [43] SNPs, with minor allele frequency above 1% and INFO score above 95% were kept for the analysis. 50 TAGR sets were used as functional categories, in addition to another set of 50 “negative TAGRs” defined excluding 500kbs around each GWAS Catalog hit.

### Models tested and covariates

We regressed each trait *t* on a linear model including the standardized *covA* for the ancestry *p* and a vector of covariates **c**:

$${t}_{i}={\beta }_{0}+{\beta }_{{{\mathrm{cov}}}A(p)}{{{\cdot }}}{{\mathrm{cov}}}A(i,p)+{{{{\boldsymbol{\beta }}}}}_{{{{\bf{c}}}}}{{{\bf{c}}}}+{\varepsilon }_{i}$$

then estimated the ${\beta }_{{{\mathrm{cov}}}A(p)}$ coefficients. Categorical traits, which were transformed to {0, 1} where 1 stands for the category described in Table S3 and 0 for all the others, were regressed with a logistic regression. We instead adopted an ordinal logistic regression for ordinal traits: Table S3 describes the category order.

The covariates included in the model are (in parenthesis the corresponding UKBB Field ID): age (21022), sex (31), age^2^, age×sex, age^2^×sex, latitude (22703), longitude (22701), UKBB assessment center (54), urban or rural home area (20118, defined collapsing codes 1,5,11,12 into “urban”; 2,6,13,14,15 into “town”; 3,4,7,8,16,17,18 into “rural” categories), qualifications (6138), age when completed education (845) and Townsend deprivation index at recruitment (22189). We also added two genome-wide *covAs* (*GW-covA*) as covariates in every model to control for genome-wide ancestry which might be passively correlated with environmental confounders. Note that given that *covA* is a relative distance, with three ancestries we only have two degrees of freedom so adding the third would have generated perfect multicollinearity. Throughout the text “*TAGR-covAs*” refers to the statistic computed on the corresponding TAGRs for each trait, while ”*GW-covA*” is the genome-wide statistic.

Then, the slope coefficient (β_*covA*_), or the Odds Ratio (OR_*covA*_) were directly used to assess ancestry-trait association for continuous and categorical traits respectively, double-sided *p*-values were computed by the glm R function. Significance was evaluated at Benjamini-Hochberg False Discovery Rate = 0.05. In all cases when reporting results for each trait we count multi-category traits only once.

When replicating our analysis using siblings we adopted the following model:

$${t}_{i}={\beta }_{0}+{\beta }_{{cov}{A}{(p)}{\hbox{'}}}{{\cdot }}{cov}{A}({i,p}){{\hbox{'}}}+{\beta }_{{cov}{A}({p}){sib}}{{\cdot }}{{cov}{A}({i,p})}_{{sib}}+{{{{\boldsymbol{\beta }}}}}_{{{{\bf{c}}}}}{{{\bf{c}}}}+{\varepsilon }_{i}$$

where *covA(i,p)*_*sib*_ is the mean *covA* for ancestry *p* of the sibship individual *i* belongs to, and *covA(i,p)’* is individual’s *i* residual so that *covA(i,p)’* + *covA(i,p)*_*sib*_ is the total *covA* value of individual *i* for ancestry *p*. It is then the ${\beta }_{{{\mathrm{cov}}}A(p)}\hbox{'}$ coefficient that we evaluate. The covariate vector includes age, sex, age^2^, age×sex, age^2^×sex and, when *covAs* are based on TAGRs, two *GW-covA(i,p)* as above.

## Results

### Simulations

We started by exploring covA strengths and limitations in the simulation framework described above. To assess the performance of *covA*, we first ranked the three reference populations (equivalent to the sampled ancient genomes used to calculate *covA*) for their mean genetic value (GV), that is the expected phenotypic value without considering the environmental deviation. Therefore in each run *p* = *1,2,3* is the population with the highest, the median, and the lowest mean GV respectively. In scenarios of directional selection *p* = *1* corresponds to the ancestry experiencing a positive shift in trait optimum. We then tracked the slope obtained when regressing the simulated trait on *covA(1)* i.e. β_*covA(1)*_.

**Fig. 1:**
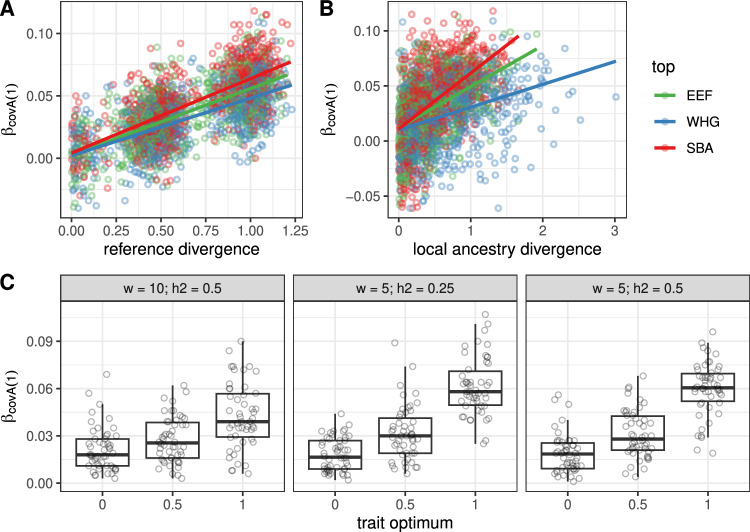
Simulation results. **A** Effect on β_covA(1)_ of reference populations differentiation, defined as the difference between the highest and the second highest mean genetic values among the three ancestries, normalized by trait standard deviation in the simulated present-day population (see Supplementary Text for details). **B** The same as **A** but plotting on x-axis local ancestry’s mean genetic values instead. **C** β_covA(1)_ values as a function of trait optimum in the ancestry with the highest genetic value (*p* = 1). The x-axis indicates the trait optimum in this ancestry, while other ancestries have an optimum set to 0. Three sub-panels present results of simulations with different fitness function SD (ω) and heritability (h^2^) values. Each boxplot is based on 50 individual simulations. Scenarios with more than 50 simulation runs were randomly downsampled. The boxes show 25th, 50th and 75th quantiles, while the whiskers show values within 1.5 times the interquartile range (IQR).

We started by verifying whether β_*covA*_ depends on the difference in the trait’s mean genetic value between reference populations. On average, higher β_*covA(1)*_ values are observed as the difference in GV between ancestries *p* = *1* and *p* = *2* increases (Fig. 1A). In other words, the more genetically differentiated are the reference populations at TAGRs, the stronger is the association between *covA* and the trait value in present-day individuals. On the other hand, we see no independent effect of heritability, polygenicity or strength of stabilizing selection on the relationship between β_*covA*_ and genetic value differentiation (Fig. S2).

Notably, we can reliably identify the reference ancestry *p* = *1* as the one with the maximal β_*covA*_ as long as it is sufficiently differentiated from the ancestry *p* = *2*: the true-positive rate is 78–87% if the difference between GV is within 0.25–0.5 of the trait’s SD in the present-day population (Fig. S3A). Note, however, that β_*covA*_ values are negatively correlated between ancestries (Fig. S4), meaning that high absolute β_*covA*_ for one ancestry will be often complemented by high absolute β_*covA*_ for at least one other ancestry but with an opposite sign.

We next asked if β_*covA*_ can be informative about differences in trait optima between ancestries. We hypothesize that drift under stabilizing selection with the same optimum in all ancestries would result in lower β_*covA(1)*_ as compared to scenarios with optimum shift in one of the ancestries. Although we do observe such a trend (Fig. 1C, S5), rather high values of β_*covA(1)*_ (97.5 percentile of 330 simulations being equal to 0.048, see Table S5) can also be obtained in simulations with constant trait optimum.

Finally, we checked whether β_*covA*_ can be informative about the differential genetic contribution of ancestral populations to present-day trait variation. Intuitively, this can be measured as the expected GV of a present-day individual with all trait-affecting loci inherited from a specific ancestry. This value might deviate from the reference population’s average GV due to post-admixture drift and selection. As can be seen in Fig. 1B and Fig. S6, β_covA_ remains informative about the difference in local ancestry GV between ancestries *p* = 1 and *p* = 2 but the true-positive rate of identifying the local ancestry with the highest GV is lower (57–78% if GV difference is within 0.25–0.5 of the trait’s SD in the present-day population; Fig. S3B). The lowest sensitivity is observed for the WHG local ancestry likely because of its low contribution to the present-day population (12%) and hence stronger effect of post-admixture drift. However, as exemplified by scenarios **E** and **F** in Fig. S6, when the reference populations had the same trait optimum, our approach is blind to differences in local ancestry genetic value arising from post-admixture drift (**E**) or selection (**F**). Full simulation results are reported in Table S1.

### Association with UK Biobank complex trait landscape

We applied the same approach to 50,000 unrelated individuals of European descent from the UKBB [2], so as to match the order of magnitude of our previous EstBB analysis [33]. The reference groups representing WHG, EEF and SBA (here defined as genetic ancestries, rather than as actual cultures or populations) included 95 WHG-, 118 EEF-, and 83 SBA-like ancient genomes, see list in Table S2, Fig. S7.

We identified TAGRs for each trait exploiting GWAS Catalog [41] hits for congruous traits (see Methods for details). Indeed, TAGRs were enriched in SNP heritability for the corresponding trait in almost all cases (stratified LD score regression [44], nominal *P*-value < 0.05, see Fig. S8A). We then computed *TAGR-covA*s for each ancestry on these regions and regressed phenotypic values for each trait on them. Notably, to capture any possible physiology-related, geographic and socio-economic confounder that might produce spurious false positives, we also include in this regression a large set of covariates: sex, age, age2, sex×age, sex×age2, latitude, longitude, UKBB assessment center, Townsend deprivation index (TDI), qualifications, and age when completed education. Importantly, we also included genome-wide *covA* for two ancestries (capturing all *GW-covA* variability) to control for genome-wide population structure.

**Fig. 2:**
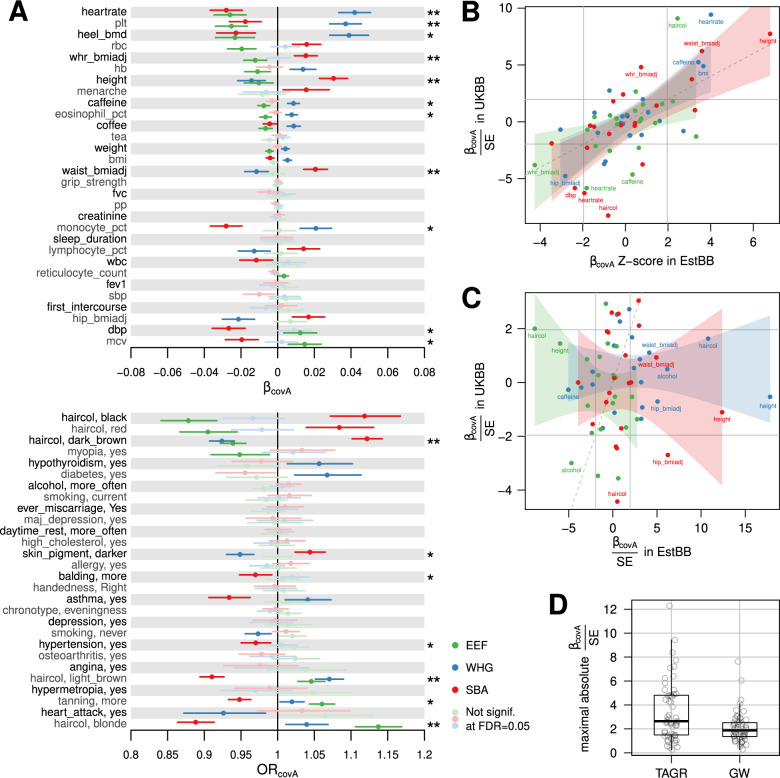
Trait-ancestry associations in UKBB and across Biobanks. **A** *CovA* effect size (β_covA_ or OR_covA_) estimates together with their 95% confidence interval, for each ancestry and for 53 traits analyzed. For each trait, *TAGR-covAs* are used in independent models as trait regressors together with several covariates (including two *GW-covAs*). *TAGR-covAs* not adding significant information according to double-sided coefficient *p*-value at 5% FDR are shown in pastel colors. Trait-ancestry associations significant and concordant in the sibling analysis are marked with one asterisk (*p* < 0.05) or two (FDR < 0.05). All β_covA_ represented in this figure, including those estimated in the sibling analysis, are reported in Table S6-7. **B** *TAGR-covA* test statistics compared between UK Biobank and Estonian Biobank. Z-scores in X axis are extracted from the original paper [33], and computed differently than β/SE. Shaded areas represent linear regression slope confidence intervals for each ancestry independently, the solid gray lines mark nominal significance boundaries for β_covA_ while the y = x line is represented as dashed. Traits showing an absolute value > 4 in any of the two axes are labeled **C** Same as **B** but here covA is computed genome-wide. **D** Maximal absolute *TAGR-covA* and *GW-covA* test statistics. Each dot is a trait.

An independent model was built for each of the ancestries as our simulations showed that a genetic value shift in a single ancestry could explain significant β_*covA*_ in all ancestries, and that the ancestry with the maximal absolute coefficient (*max(|*β_*covA*_ | *)*) is likely to be the most divergent one. As seen in Fig. 2A, 32 out of 53 traits tested show at least one significant β_*covA*_ (or OR_*covA*_) at 5% Benjamini-Hochberg False Discovery Rate (FDR). Affected traits include several biological domains and will be addressed in the last results section. Notably, no trait exhibits a *max(|*β_*covA*_ | *)* > 0.048, which based on our simulation results would have been a strong indication of pre-admixture selection.

### Assessing the robustness of TAGR-covA effects

Although we include multiple covariates in our model, and we show that there is no strong collinearity between them and TAGR-covA (see Supplementary Text), we wanted to further ensure that we are not detecting associations between traits and *covA* due to unaccounted environmental confounding. We therefore aimed at validating our results by using trait and *covA* differences between siblings within UKBB (see Methods for a description of the model). Although siblings might differ in their environment, those differences do not correlate with genetics due to population structure. Hence, this approach is robust to confounding due to overlapping genetic and environmental stratification. 16 out of 32 traits significant in our main analysis were recovered with nominal significance and concordant effect direction in at least one ancestry (Fig. 2A). We observe a general effect concordance (no significant effects in the opposite direction, see also Fig. S9): most of the missing signals can be explained by a lower statistical power.

Next, for 21 traits already explored in the Estonian Biobank (EstBB) [33] we compared the regression statistics obtained across biobanks, finding a remarkable consistency, see Fig. 2B. Even though we refrain from assigning a p-value due to the plausible correlation across several trait pairs, we identify a Pearson’s correlation coefficient between biobanks of ρ = 0.729, ρ = 0.542, and ρ = 0.726 respectively for WHG, EEF, and SBA ancestries.

We repeated the analysis by excluding *TAGR-covA*s and instead evaluating the coefficients of *GW-covA*. These were previously used as genomic control covariates, thus allowing us to only keep physiology-related, geographic and socio-economic factors as covariates this time. In this setting we can still see 11 out of 53 significant signals, see Fig. S10, seemingly suggesting that the global similarity with a specific ancestry could be associated with a certain trait. However, the signals are often discordant and have lower absolute effect size on average: for above-mentioned reasons we refrain from conducting a formal test but the trend is appreciable in Fig. 2D. Moreover, the consistency with the sibling analysis and across biobanks is completely absent if we compare *GW-covAs* (Fig. S9 and Fig. 2C, respectively) suggesting that these signals are likely resulting from yet unaccounted confounders. Indeed, although likely harboring variants contributing to the polygenic traits analyzed, the remaining non-TAGR genome was often depleted in trait heritability (see Fig. S8B) but equally subject to confounders impacting at genomic level.

### Ancestry-specific diverging traits

We interpret significant β_*covA*_ as ancestry divergence in genetic value and designate the genetic component showing max(|β_*covA*_ | ) as the most divergent one. Note, however, that in opposition to our simulations, where only one ancestry was divergent by design, the ancestry with max(|β_*covA*_ | ) might not be the only one that experienced a shift in GV. In the following description, we will privilege associations validated in our sibling analysis, but all significant associations in Fig. 2A could in theory be validated with a larger sibling sample size.

Resting heart rate, platelet count and heel bone mineral density, which increase together with WHG ancestry similarity, show the highest absolute β_*covA*_. As shown by our simulations, adaptation might not be necessary to observe such divergence. Nevertheless, given health-relevant changes in platelet count and heart rate variability during winter [45, 46], coupled with the complex heart rate reactions to cold exposure [47, 48], it is tempting to speculate about an adaptation to colder climates that WHG suffered when colonizing Europe soon after the last glacial maximum (after 17 kya [49]). Interestingly, platelet count was found to be selected for lower levels since the early Neolithic [50, 51] thus supporting the idea of higher values being WHG-specific and possibly maladaptive in environments tied to non-hunter-gatherer lifestyles.

We replicate known signals of genetic divergence across ancestries for anthropometric and pigmentation traits [33], most notably an association of SBA ancestry with tall stature [26], large waist and waist/hip ratio, but also with darker hair and skin pigmentation, mirrored by opposite effects associated with EEF component. This agrees with ancestry-specific risk scores for these traits estimated by Irving-Pease et al. [29], considering that our SBA component should conflate their Caucasus Hunter-Gatherers, Eastern Hunter-Gatherers and Steppe ancestries. Overall, these results corroborate the hypothesis that pigmentation decrease in Europe is due to post-admixture selection rather than to the impact of incoming SBA migrations during the Bronze Age [27, 50].

For some traits such as caffeine intake, as well as for previously mentioned traits (e.g. waist-derived traits, pigmentation), two ancestries are both significant for opposite trait values. This is expected due to the negative correlation between β_*covA*_ (Fig. S4) which is given by design but can be further enhanced by divergent evolution of the two ancestries. In some scenarios, only one directional association is replicated by the sibling analysis, resulting from the lower statistical power of this analysis: this is the case for eosinophil percentage, hypertension prevalence and ease of tanning with EEF remaining significant; and monocyte percentage, mean corpuscular volume, diastolic blood pressure and balding with SBA as the validated component.

## Discussion

We applied *covA* to the largest European biobank to identify which complex trait population gradients can be explained by genetic similarity with one of the three main European ancestries: WHG, EEF, and SBA. In our work, we found that relative similarity with a specific ancestral component at trait-associated genomic regions significantly explains a portion of the variance in 32 out of 53 complex traits.

Our findings are globally consistent with an independent analysis conducted on the Estonian Biobank for 21 overlapping traits, thus confirming that these results should be taken as indicative of continental patterns rather than just regional ones. Most of these associations were also confirmed in sibling analysis thus providing evidence against these findings being due to unaccounted environmental confounders conflated with population structure.

Noteworthy, whole-genome results were inconsistent across biobanks, unrelated vs. sibling analysis, and when compared with TAGRs results within the same dataset (Fig. 2C, D, S9); in general whole-genome ancestry similarity was a poor trait predictor. In other words: although we took measures to control for confounders by including multiple geographic and socio-economic variables into our models, only by enriching for biologically-relevant regions, and by controlling for *GW-covA* stratification, we could expose robust and consistent signals. This finding mirrors the known difficulty in discerning functional gene-trait associations from spurious correlations mediated by genetic structure [16–18, 20, 21], which are indeed independent between different populations and different biobanks with different recruitment strategies. In addition, while combining GWAS-derived effect sizes at the genome scale is sensitive to subtle correction-surviving biases [19], our approach does not rely on it. Our results are partially overlapping with an independent investigation with a different approach [29], where a different choice of source ancestral groups and use of GWAS-derived weights for the trait-associated genetic variants may explain emerging differences.

While we are confident about the robustness and the continental scope of the results presented in Fig. 2A, the biological and evolutionary dynamics that can explain these associations remain rather complex to establish. We thus complemented the empirical analysis with extensive simulations to provide insights useful in interpreting our results.

Specifically, the simulations show that significant β_covA_ for at least one of the ancestries can be most directly interpreted as differences in genetic values between sampled reference populations. Although in our simulations such differences can be reached under scenarios with all reference populations having the same optimum, scenarios with optimum shift in one of the ancestries in general result in higher absolute β_covA_ (Fig. 1C) for that ancestry. As the highest β_covA_ values we observe on the real data are close to the 97.5th percentile of the β_covA_ distribution under equal optima in simulations, it is likely that the top of our list contains at least some traits that underwent directional selection in different ancestral populations. Indeed, our results highlight that resting heart rate, blood platelet count and bone mineral density present divergence patterns compatible with adaptive selection. These patterns are comparable to those observed in extensively studied traits like pigmentation and height, warranting a similar level of investigation.

Our simulation results also highlight the potential limitations of our study. These mostly relate to interpreting β_covA_ as an indication of the differential genetic contribution of ancestries to trait variation in the present-day population. On the one hand, such contribution can be overlooked if genetic differentiation between ancestries post-dates the available aDNA samples used as references (Fig. S6E). Furthermore, as most common alleles are expected to pre-date the split between the three ancestries, present-day individuals with higher GV for a given trait might show higher *covA* with an ancestry enriched for trait-increasing alleles, even without any direct genetic contribution from that ancestry. Thus, choosing and exhaustively including all relevant ancestral groups is crucial for a sound biological interpretation of *covA* signals. This limits the applicability of our approach to populations for which the demographic history is less well understood and proper reference aDNA samples are missing.

Finally, two aspects need to be emphasized. First, covA effect sizes were almost never above 0.1, even in the most favorable simulation scenarios: this means that the majority of trait variation should be traced to genetic variation common across ancestral groups, or to environmental effects. Second, the differences in genetic predispositions between ancestries to the tested phenotypes emerge from contemporary phenotypic landscapes and might involve complex interactions with the environment thus weakening claims about actual trait values in the ancestral populations.

In conclusion, the reported ancestry-trait associations are strongly indicative of ancestry-specific genetic predispositions, possibly due to pre-admixture selection, and under certain conditions indicate actual genetic contribution to the contemporary trait landscape. Especially for the most significant associations described above, it is important to stress how the European population has to be assumed as inherently stratified due to its demographic history. This bias will need to be addressed in analyses following up on GWAS effect sizes, in order to avoid potential spurious results. This stratification acts therefore both as a source for variability in such traits and as a potential significant confounder for GWAS study across European cohorts.

## Supplementary information

Supplementary Materials

Supplementary Tables

## Data Availability

UK Biobank data can be accessed through their procedure including project ethical assessment and access fees. Ancient DNA data from Allen Ancient DNA Resource is available at https://dataverse.harvard.edu/dataset.xhtml?persistentId=doi:10.7910/DVN/FFIDCW. All produced results will be available as supplementary materials or through an online GIT repository.

## Code availability

Simulations: https://github.com/vasilipankratov/SLiM_quantitative_trait. All remaining code will be available as an online GIT repository upon acceptance.
