# Supplementary material for: Ancestral genetic components are consistently associated with the complex trait landscape in European biobanks
Source: Eur J Hum Genet. 2024 Aug 10;32(11):1492–9. doi: 10.1038/s41431-024-01678-9 (PMC11576899; doi:10.1038/s41431-024-01678-9)
Supplement: Supplementary file 1 — Supplementary Materials [file 41431_2024_1678_MOESM1_ESM.pdf]

Supplementary Material for:

Ancestral genetic components are consistently associated with the complex trait landscape in European Biobanks

Vasili Pankratov, Massimo Mezzavilla, Serena Aneli, Ivan A Kuznetsov, Daniela Fusco, James F Wilson, Mait Metspalu, Paolo Provero, Luca Pagani, Davide Marnetto

Supplementary Text

Simulations Supplementary Methods

To decrease runtime we split the simulation into three steps described in **Table S8**.

Each subsequent step takes the output of the previous one as input and inherits the recombination and mutation rates. Where appropriate, multiple instances of subsequent steps started from the same output of the previous step to save run-time and disc space.

Step 1 was performed in msprime v1.2.0[1] using the discrete time Wright-Fisher model[2]. We simulated 1000 unlinked genomic regions each being 20 kb long with a uniform recombination rate of 1e-8 and uniform mutation rate of 1.25e-8 in a panmictic population with a constant effective population size of 14,000. The pyslim v1.0.1 package was used to convert the msprime's tree sequence object into a SLiM sim object.

Step 2 was run in SLiM v4.0[3] simulating a panmictic population of 14,000 diploid individuals. At the very beginning of this step we randomly picked 1000 genetic variants (one per each genomic region) meeting the following criteria: a) maf >= 0.1 at the beginning of step 2; b) position in the genomic region is between 5 and 15 kb. Each of the 1000 variants was then randomly assigned an effect size from either a single or a mixture of two zero-centered normal distributions (**Table S9**). In the latter case (setup 3 in **Table S9**) where we simulate lower trait polygenicity (i.e. a small fraction of variants having strong effect on the trait) we sampled the effect sizes from a high variance distribution (a) with probability 0.05 and from a low variance distribution (b) with probability 0.95. Effect sizes are constant across generations and populations hence no gene-by-environment interactions are simulated. All mutations originating during steps 2 and 3 are assumed to have no effect on the trait.

Step 3 was implemented in SLiM v4.0[3]. The modeled demography is described in Figure S1 and **Table S10.** Specifically, we model the British population as a result of admixture that took place 140 generations ago between Late European Farmers, LEF (54%) and Steppe Bronze Age (46%) ancestries. In turn, Late European Farmers are modeled as a result of admixture between Early European Farmers, EEF (78%) and Western Hunter-Gatherers, WHG (22%) 270 generations ago. The British population started growing exponentially 17 generations from Ne of 6000 at a rate of 0.18 reaching Ne of approximately 100K by the end

of the simulation. Although according to IBD-based estimates[4] the present-day Ne of the British population might be on the order of 10M we aim our simulation at 100K for performance reasons. The sampled ancient genotypes come from reference populations that diverged from the populations actually involved in the admixture events (EEF, WHG and SBA) 50 generations before the admixture.

To explore the effects of directional selection we modeled trait optima shifts in one of the following populations at a time: EEF, WHG, SBA. The optimum was either kept constant at 0 in all populations (no directional selection) or shifted to +0.5 or +1 depending on the specific simulation. The optimum shift is implemented right after the origin of the corresponding population (**Table S10**). This results in different populations having different amounts of time to adapt to the new optimum.

When populations differed in their optima we explored the following approaches to model the trait optimum of admixed populations, resulting in different post-admixture selection regimes:

1. The optimum of an admixed population is a weighted average of the two admixing populations optima. For instance, if the optimum of EEF is 1 and the optimum of WHG is 0, the optimum in LEF is 0.78.
2. The optimum of an admixed population is the maximum of the two admixing populations optima. For instance, if the optimum of EEF is 1 and the optimum of WHG is 0, the optimum in LEF is 1.
3. The optimum of an admixed population is twice the maximum of the two admixing populations optima. For instance, if the optimum of EEF is 1 and the optimum of WHG is 0, the optimum in LE is 2.
4. The optimum of an admixed population is the minimum of the two admixing populations optima. For instance, if the optimum of EEF is 1 and the optimum of WHG is 0, the optimum in LEF is 0.

We additionally modeled trait optimum equal to +2 in the UK or the LEF populations. See **Table S11** for more details.

In steps 2 and 3 genetic value for each individual in each generation was calculated by summing up counts of alternative alleles at each causal variant weighted by the corresponding effect size. The trait value is calculated by adding a random variable from a normal zero-centered distribution to the genetic value, mimicking environmental noise (**Table S9**). The trait is measured in arbitrary units, however, by design its standard deviation in the simulated present-day population is mostly within the range between 0.9 and 1 (Figure S11).

The fitness of each individual is calculated based on its trait value using the following formula:

$$Fitness = e^{-\frac{(opt - t)^2}{2\times\omega^2}}$$

where *opt* is the trait optimum of the population, *t* is the individual's trait value and ω the standard deviation of the fitness function; higher ω values correspond to weaker stabilizing selection.

The latter may take one of the three values: 3, 5 and 10. The choice of those values is inspired by Sanjak et al., 2018[5] who proposed the interquartile range of ω for present-day human traits to be 5.3 - 13.3 and we assume these values to be lower in ancient populations. Another simulation study[6] used ω values of 4, 5 and 7 so our range is fully overlapping but wider. All those parameters stay constant across generations (including phase 3) and populations.

As a result of both random assignment of effect sizes and changes in causal variants allele frequencies due to drift, stabilizing and directional selection (in step 3) heritability of the selected trait can differ both between simulations and through time in a given simulation (**Table S12**).

We sampled 10K genotypes and corresponding trait values from the present-day British population at the end of the simulation and 100 genotypes from each of the three reference populations: EEF, WHG and SBA at the time of the corresponding admixture events. The UK samples were pruned to keep only 1 individual out of each pair with first or second degree relatedness, resulting in around 7K individuals remaining.

For *covA* calculation we kept only regions with the causal variant effect size meeting the following criteria:

$$\frac{2\beta^2 f(1-f)}{\sigma_t^2} > 1.5 \cdot 10^{-5}$$

where β is the true effect size on the trait, *f* is derived allele frequency and σ_t^2 is the phenotypic variance in the present-day population. The threshold of 1.5e-5 corresponds to an average expected p-value = 5e-8 in a GWAS for a continuous trait with a sample size of 200K. This was done to imitate GWAS discovery and resulted in a median of 495 regions used in *covA* calculation (min = 351, max = 565).

Exploring collinearity between covA and covariates

We checked whether our results could have inflated variance due to multicollinearity with the large set of covariates. We computed Variance Inflation Factor (VIF) for β_{covA} in all models, excluding from this evaluation ordinal traits, for which VIF estimation is less straightforward. The largest VIF is 1.795, obtained for $\beta_{covA(EEF)}$ when modeling height, which is well below what is expected in case of multicollinearity, I.E. above 2.5-10 depending on conservativity choices. Indeed, the coefficients for adopted covariates in a model that does not include TAGR-covA never deviate by more than 0.121 standard errors (SEs) from the model described above, while being predictive for many traits (**Figure S12A-B**). GW-covAs are an exception, but being computed on the whole genome, they are trivially correlated with TAGR-covAs for increasingly large TAGRs, albeit not enough to trigger instability in effect size estimates (see **Figure S12C**).

Supplementary Figures

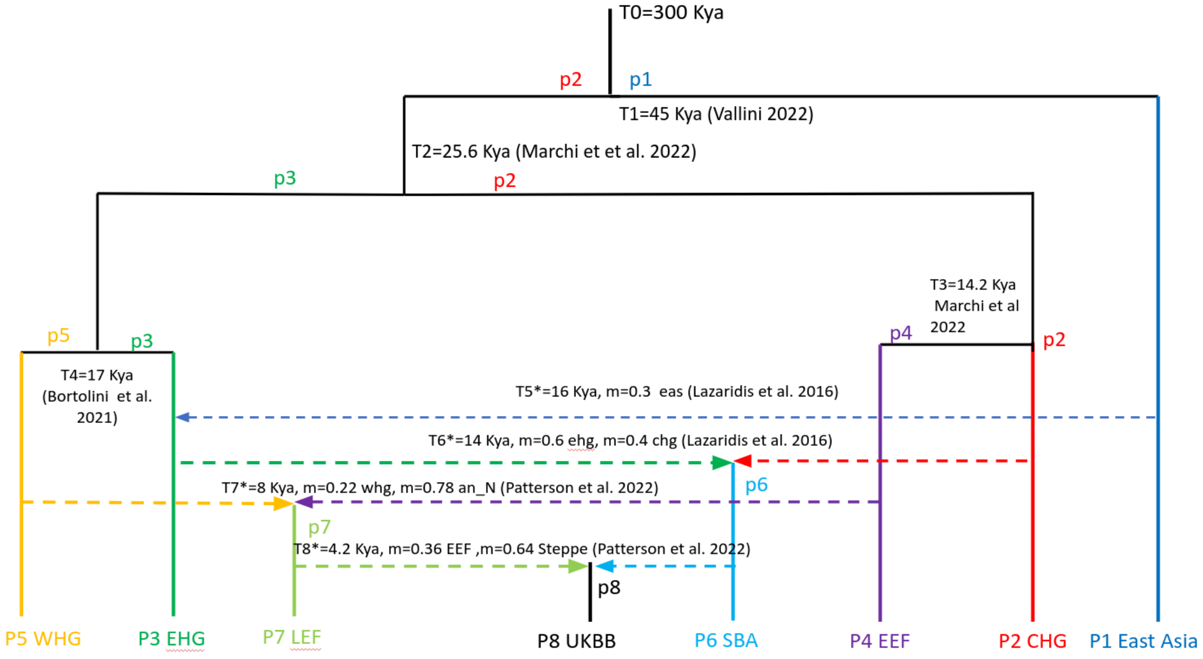

Supplementary Figure 1. *The demographic scenario used for the simulation framework. Admixture and migration events are characterized by the symbol *. The code for the populations is the following: WHG (Western Hunter-Gatherers), EHG (Eastern Hunter-Gatherers), LEF (Late European Farmers), UKBB (UK Biobank present-day individuals), SBA (Steppe Bronze Age), EEF (Early European Farmers), CHG (Caucasus Hunter-Gatherers) and East Asia. In the graph we report the estimated time for each event (split, admixture, migration) measured in thousands of years according to the literature, as follows: T1 - Vallini et al 2022[7]; T2,T3 Marchi et al 2022[8]; T4 - Bortolini et al 2021[9] ; T5,T6 - Lazaridis et al 2016[10]; T7,T8 - Patterson et al. 2022[11]*

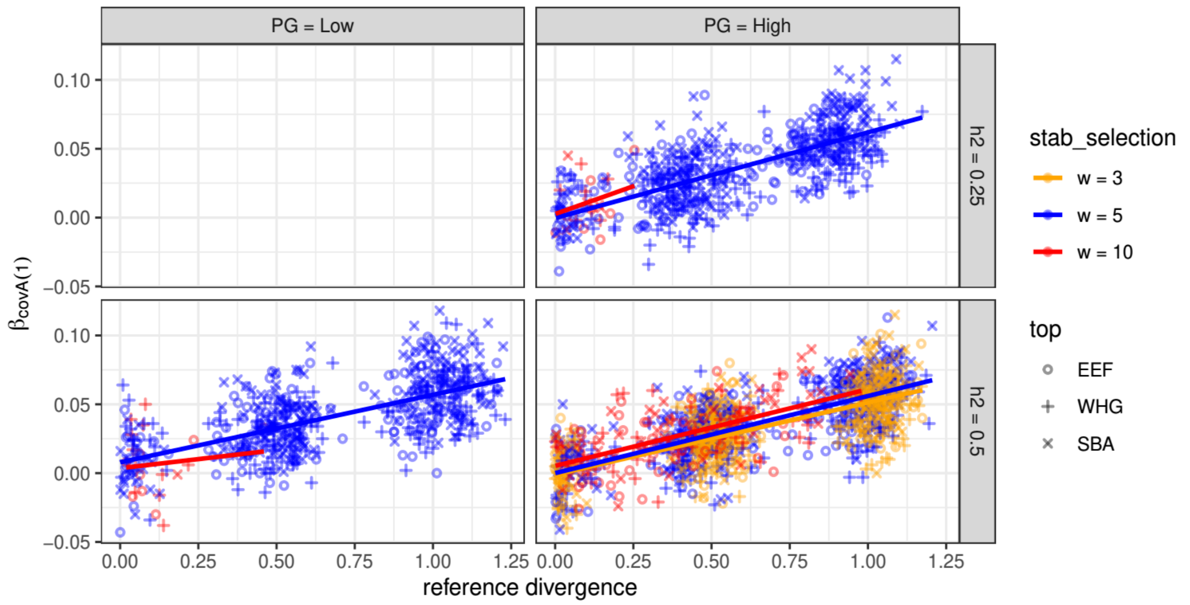

Supplementary Figure 2. *Effects of stabilizing selection strength ("stab_selection"), heritability ("h2") and polygenicity ("PG") on the relationship between beta lm(trait~covA) and genetic value differentiation. As in Figure 1 in the main text, each data point corresponds to a single simulation and reports the beta for the ancestry with the highest genetic value ("top"). Scenarios with h2 = 0.25 and PG = low were not tested.*

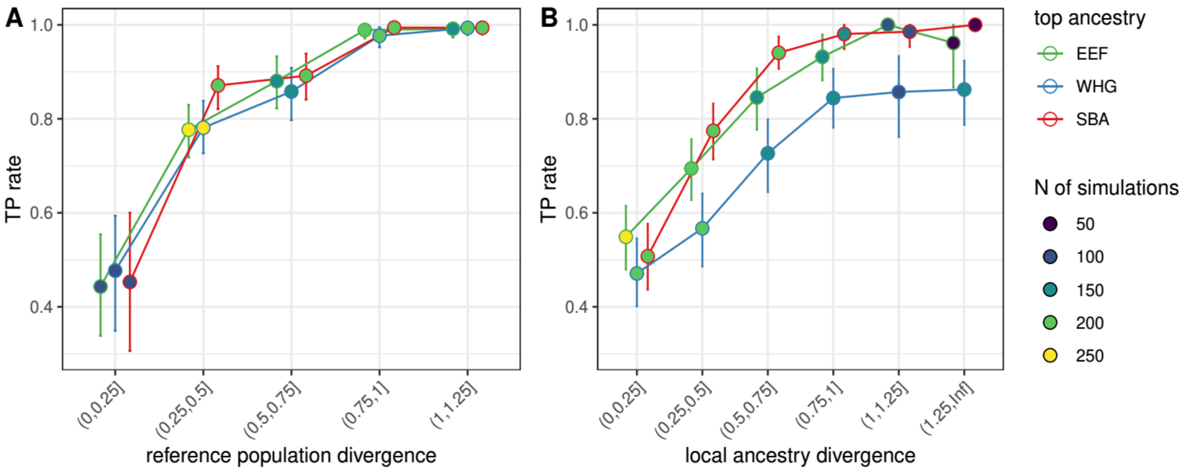

Supplementary Figure 3. *Fraction of simulations with the ancestry with the highest* β_{covA} *("top ancestry") having also the highest mean genetic value among reference populations (A) or local ancestries in the contemporary genomes (B) depending on the level of ancestry differentiation (defined as in Figure 1 in the main text). The whiskers represent 95% confidence intervals obtained by bootstrapping simulations 1000 times.*

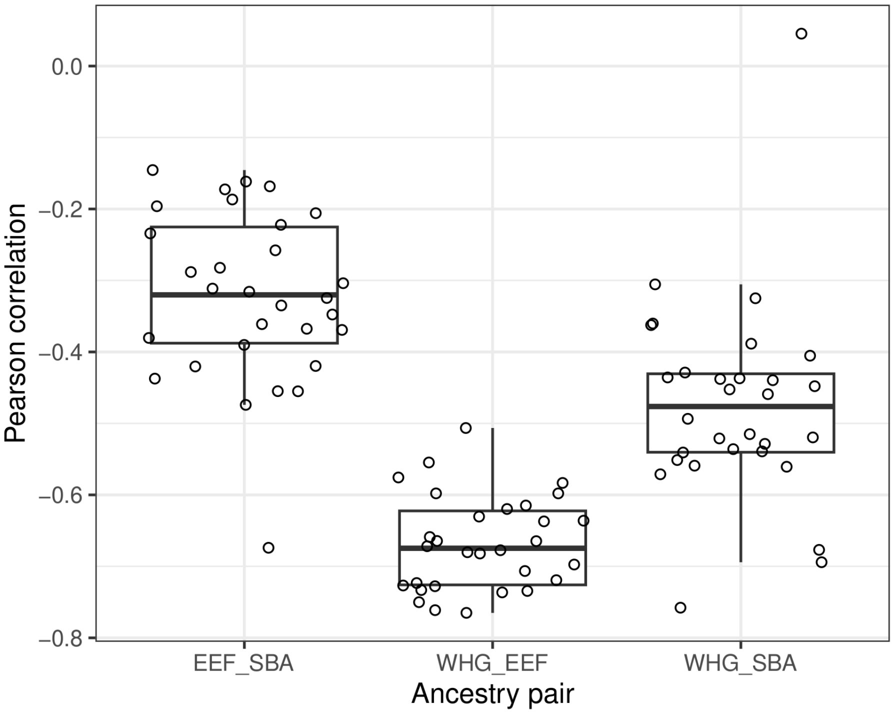

Supplementary Figure 4. *Correlation between $\beta_{covA(j)}$ for different ancestries. Each data point on the plot is a Pearson's correlation coefficient between $\beta_{covA(j)}$ for the two specified ancestries under a specific simulation scenario. Each correlation estimate is based on at least 80 runs. See **Table S13** for details.*

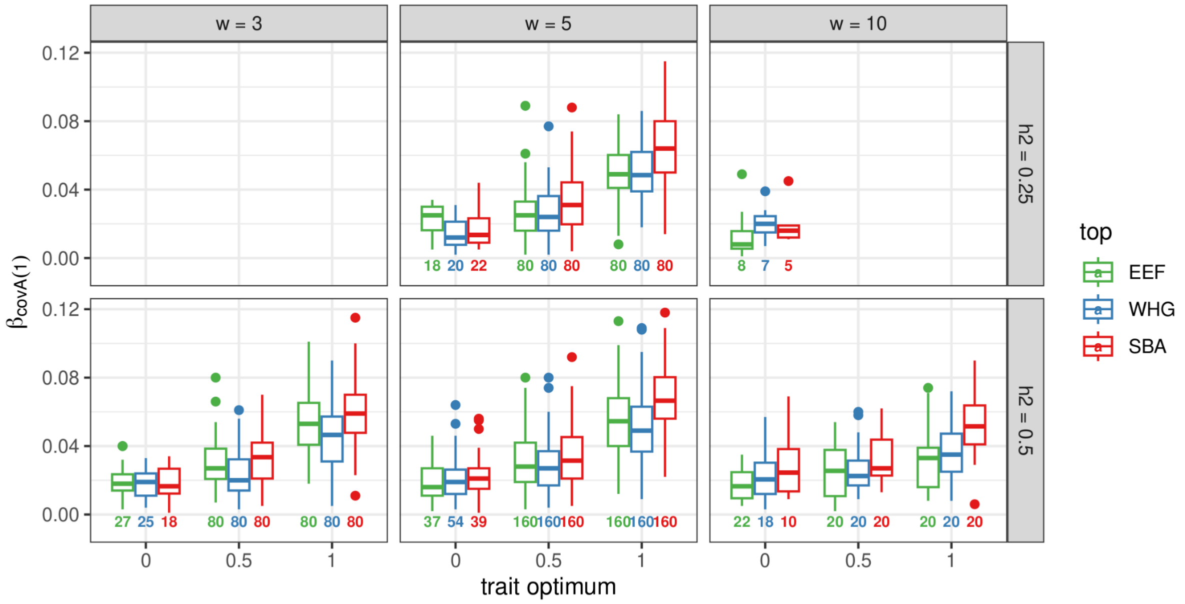

Supplementary Figure 5. *Distribution of beta values for the ancestry with the highest genetic value (which always matches the ancestry that underwent a shift in the trait optimum in scenarios where such shift was modeled) in scenarios with all three reference populations having the trait optimum at zero ("0" on x-axis) or one of the ancestries experiencing an increase of the optimum by 0.5 or 1 arbitrary units. For reference, trait sd in the contemporary population is mostly within the range between 0.9 and 1 (Figure S11). The boxes show 25th, 50th and 75th quantiles, while the whiskers show values within 1.5 times the interquartile range (IQR).*

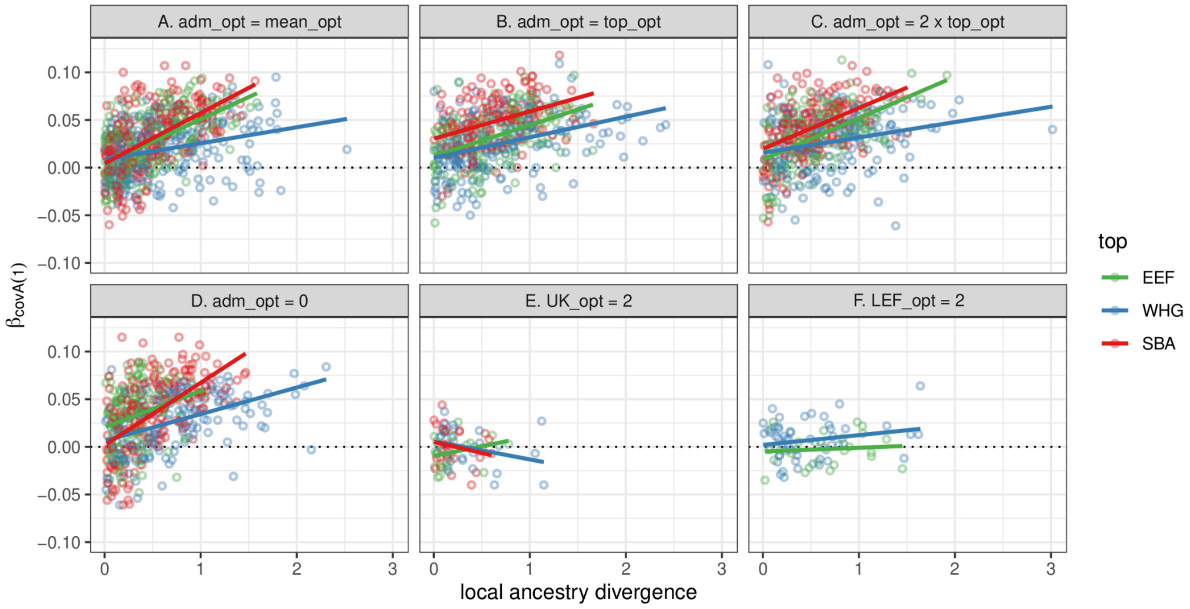

Supplementary Figure 6. *Relationship between lm(trait~covA) beta and local ancestry divergence, defined as the difference between the highest and the second highest mean genetic values among the three local ancestries, normalized by trait standard deviation in the present-day population. See Simulations Supplementary Methods for a detailed description of the different scenarios of post-admixture selection (A-F).*

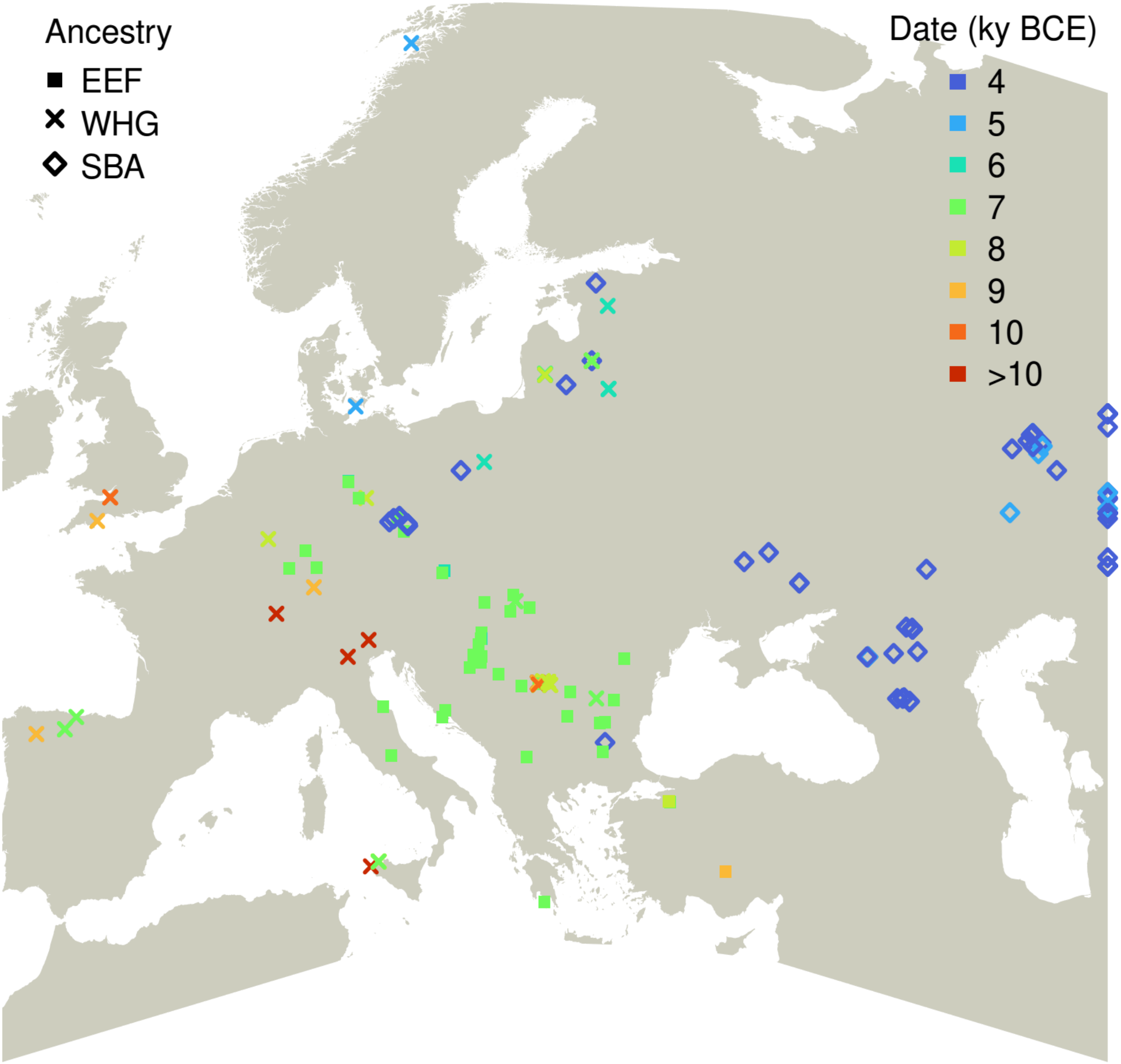

Supplementary Figure 7. *Geographical locations of samples assigned to the three ancestral groups considered, with color coding for their average date estimate in thousand years Before Current Era.*

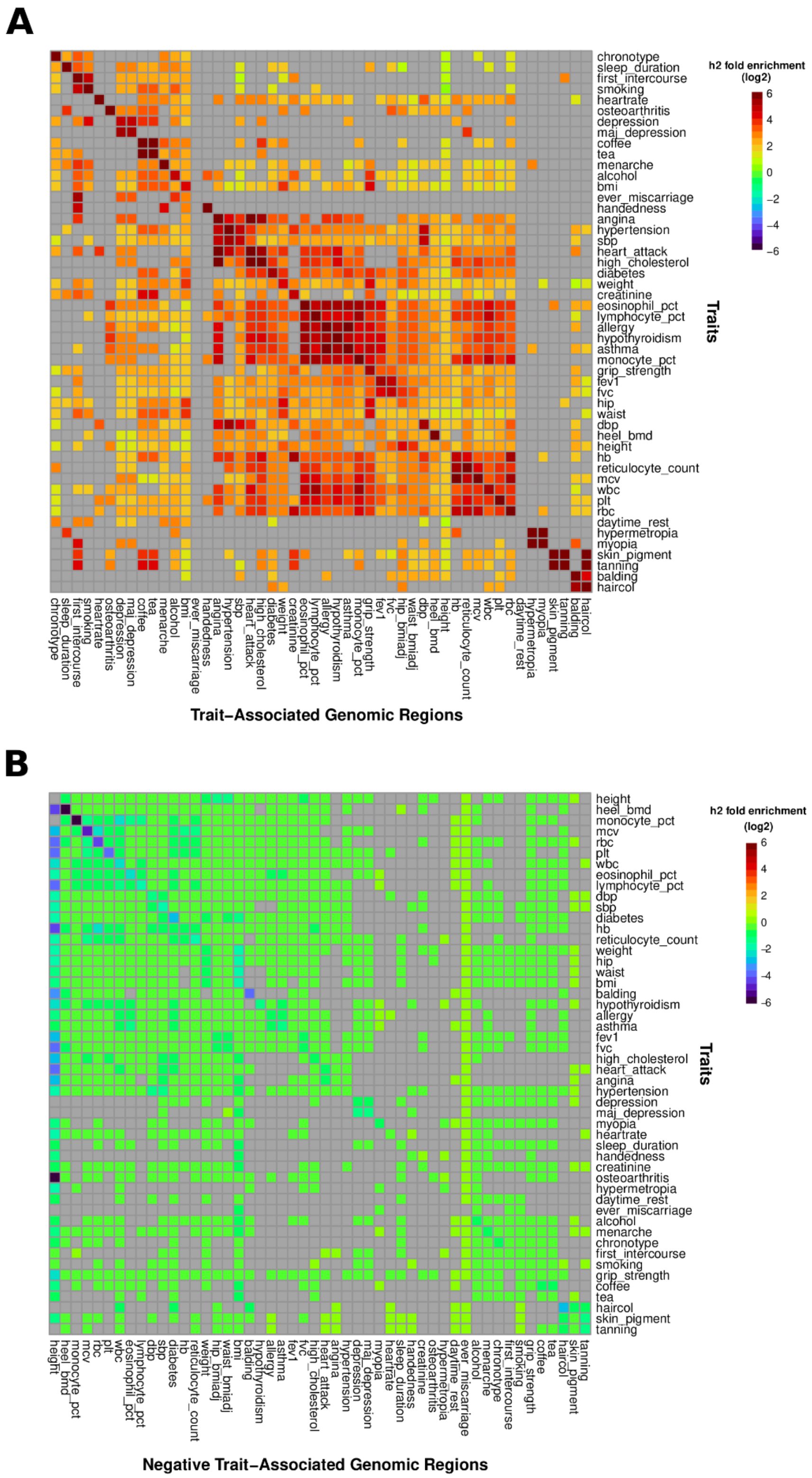

Supplementary Figure 8. *Heritability enrichment estimates for (A) TAGRs (50 annotations), and (B) negative TAGRs, defined excluding GWAS hits. Enrichment of traits expressed as (Proportion of heritability)/(Proportion of SNPs). All colored cells indicate nominal significance at P<0.05, while gray cells are not significant. Annotations are ordered by clustering. Overall, all traits show their highest enrichment in correspondent genomic regions.*

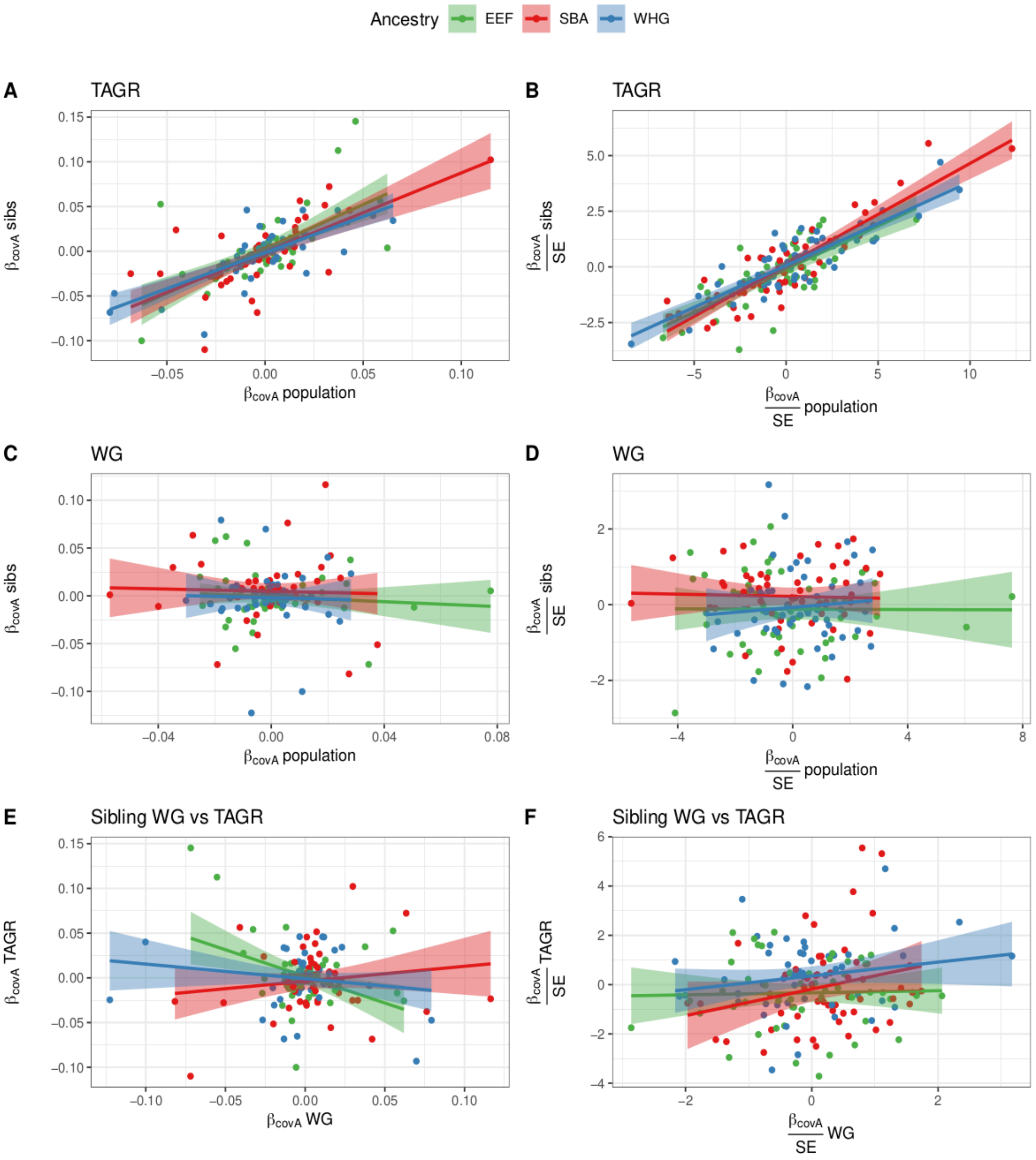

Supplementary Figure 9. Here we compare TAGR (panels A and B) and GW (panels C and D) results between the sibling-based and the population-based analyses, as well as comparing sibling-based GW to sibling-based TAGR results (panels E and F) for 53 traits for each of the three ancestries. In panels A, C and E we compare estimates of *covA* effect sizes while in B, D and F we compare *covA* effect sizes normalized by the standard error. For categorical non-binary traits we kept only the category with the lowest p-value in sibling-based TAGR analysis: for smoking this is 'never' and for hair color, this is 'dark_brown'.

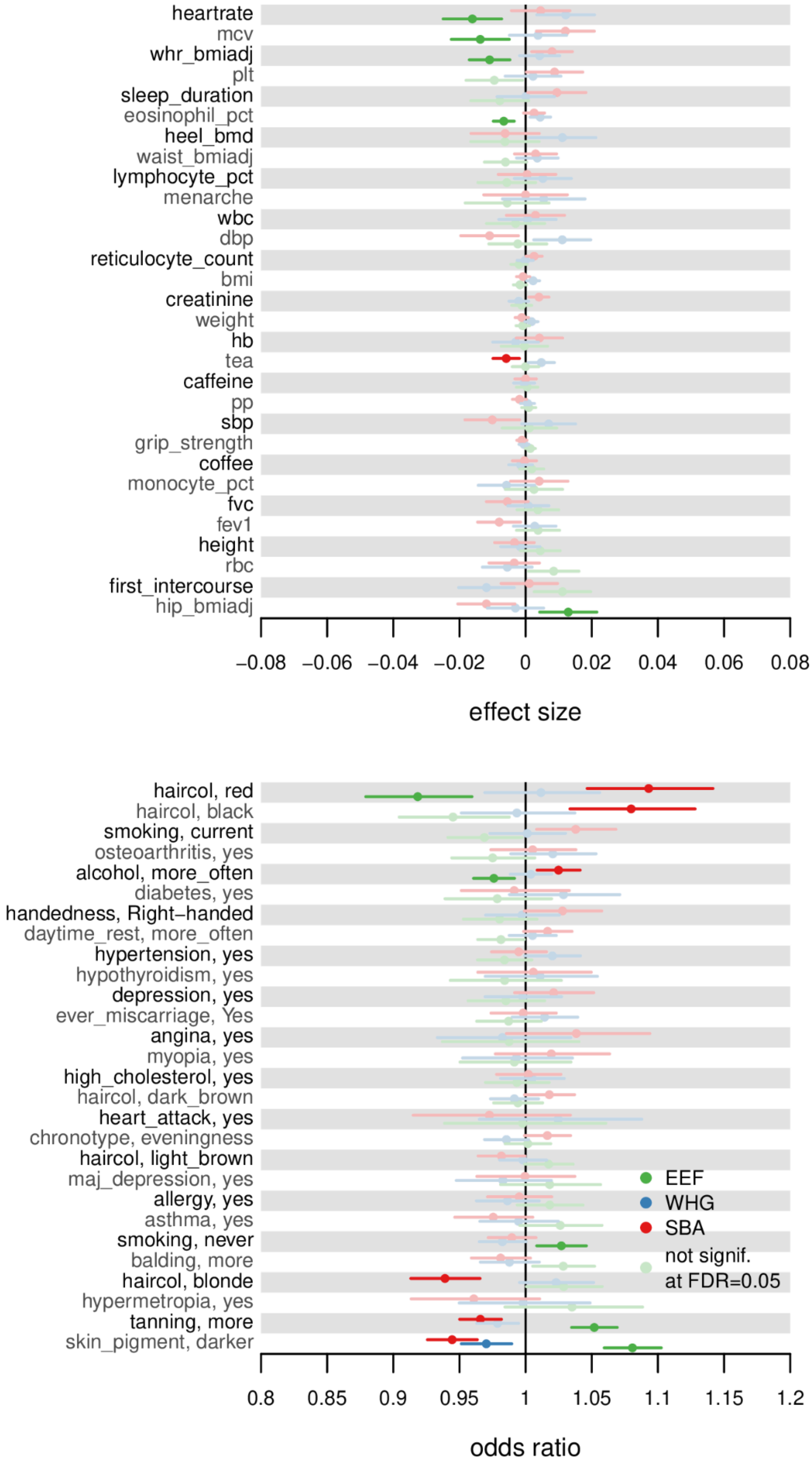

Supplementary Figure 10. *Trait-ancestry associations in UKBB using genome-wide covAs (GW-covA). Besides the origin of the covA metric, the figure details correspond to what shown in Figure 2A in the main text.*

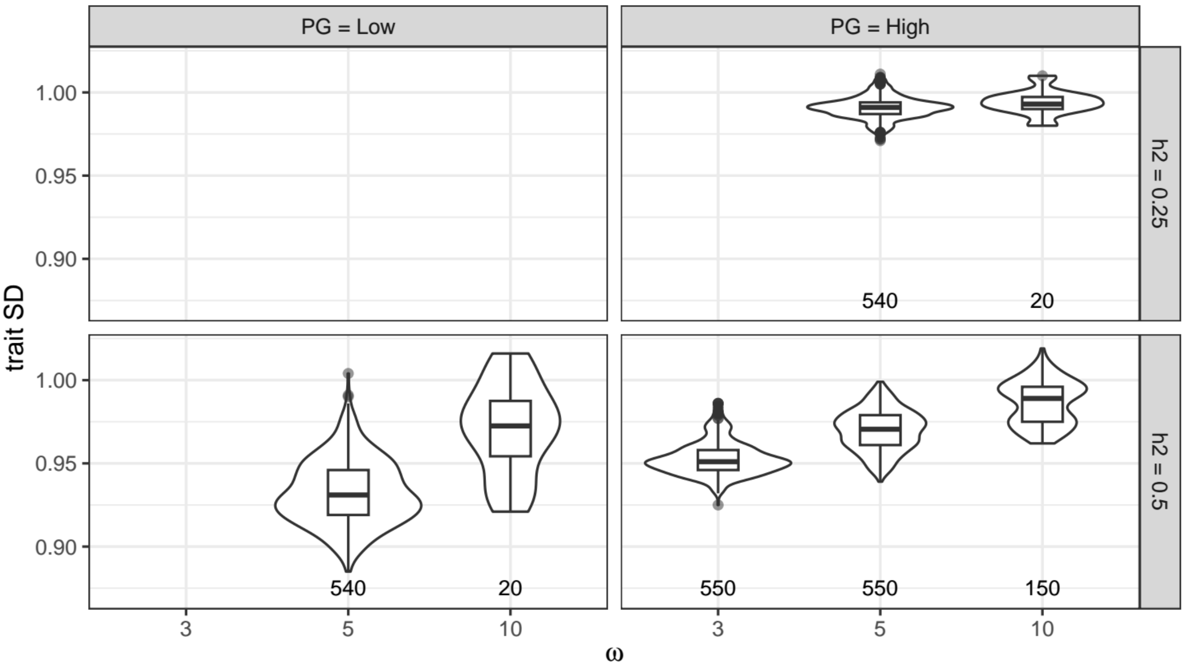

Supplementary Figure 11. *Trait standard deviation in the simulated contemporary population for various values of polygenicity (PG), heritability (h2) and stabilizing selection (expressed as the standard deviation of the fitness function, w). Numbers below the boxplots show the number of simulations in each category. Scenarios with h2=0.25 and PG=Low were not tested. The boxes show 25th, 50th and 75th quantiles, while the whiskers show values within 1.5 times the interquartile range (IQR).*

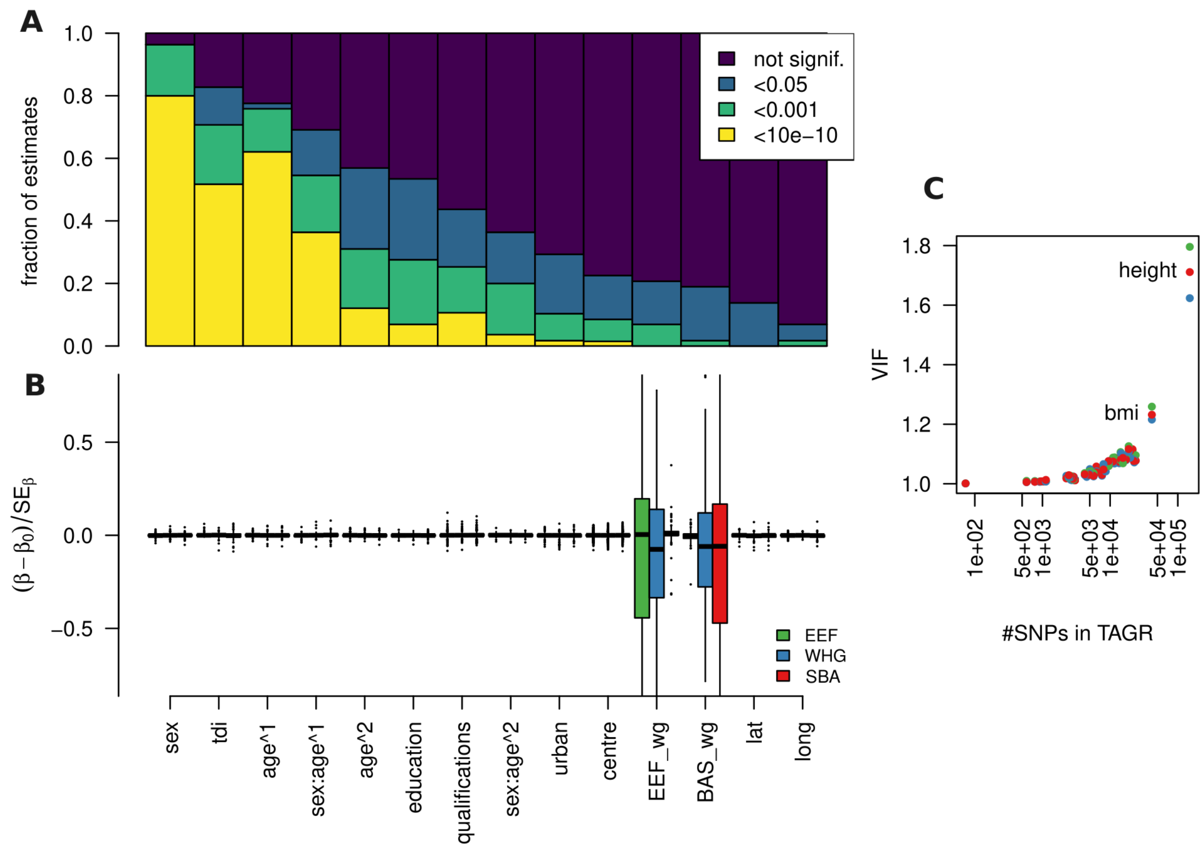

Supplementary Figure 12. *Covariates effects and multicollinearity. (A) We report the significance of covariate effect estimates across all traits in a base model not including TAGR-covA (β_b). For categorical covariates, estimates for all categories are pooled together. (B) For the same covariates we show the estimate difference in SE_β when adding the TAGR-covA to the base model, where β is the coefficient estimated in this way while β_b* is the coefficient estimated in the base model. Differences are stratified for the *covA* ancestry included in the model (C) *TAGR-covA Variance Inflation Factor (VIF) as a function of TAGR size measured in SNP count. Colors represent the covA ancestry included in the regression, with the same color key in (B).*

Supplementary Tables

Supplementary Table 1. *Simulation results (see supplementary spreadsheet).*

Supplementary Table 2. *Classification of ancient samples into ancestral groups, together with geographical coordinates, dates and PCs used for classification (see supplementary spreadsheet).*

Supplementary Table 3. *Traits description, sample sizes and transformation (see supplementary spreadsheet).*

Supplementary Table 4. *GWAS Catalog search terms for the definition of TAGRs (see supplementary spreadsheet).*

Supplementary Table 5. *β_{covA} confidence intervals in scenarios with all three ancestries having the same trait optima. To explore the variability in the results due to pulling together runs with different ω and h2 obtained the CI for different simulation groups. Each simulation group includes all simulation runs with specific combinations of ω and h2 listed in the second column.*

Simulations group	*Combined scenarios (based on ω and h2)*	*N of simulations*	*q2.5*	*q97.5*
1	*w=3; h2=0.5<br>w=5; h2=0.25<br>w=5; h2=0.5<br>w=10; h2=0.25<br>w=10; h2=0.5*	*330*	*0.0030*	*0.0483*
2	*w=5; h2=0.25<br>w=5; h2=0.5<br>w=10; h2=0.25<br>w=10; h2=0.5*	*260*	*0.0030*	*0.0500*
3	*w=5; h2=0.25<br>w=5; h2=0.5<br>w=10; h2=0.5*	*240*	*0.0030*	*0.0501*
4	*w=5; h2=0.25<br>w=5; h2=0.5*	*190*	*0.0030*	*0.0471*
5	*w=10; h2=0.25<br>w=10; h2=0.5*	*70*	*0.0037*	*0.0519*

Supplementary Table 6. *β_{covA} estimates and their significance derived from all TAGR- and GW-covA models in unrelated samples (see supplementary spreadsheet).*

Supplementary Table 7. *β_{covA} estimates and their significance derived from all TAGR- and GW-covA models in siblings (see supplementary spreadsheet).*

Supplementary Table 8. *Simulation steps.*

Step	Simulator	Duration	Description
1. Neutral burn-in	msprime	Till coalescence	Generating genetic diversity at mutation-drift equilibrium
2. Burn-in with stabilizing selection	SLiM	1000 generations	Picking causal variants, assigning effect sizes to those and equilibrating genetic variance to stabilizing selection
3. Main simulation	SLiM	1500 generations	Simulating all demographic events, directional selection and sampling genomes and phenotypes

Supplementary Table 9. *Distributions used to draw variant effect sizes and environmental noise values.*

Setup	Polygenicity	Target h2	Effect size variance	Environmental noise variance
1	High	0.5	1.5/1000	0.5
2	High	0.25	0.75/1000	0.75
3	Low	0.5	a) 0.5*1.5/50 with prob 0.05<br>b) 0.5*1.5/950 with prob 0.95	0.5

Supplementary Table 10. *Description of the simulated demographic history during step 3 of the simulation (see supplementary spreadsheet).*

Supplementary Table 11. *Number of simulation runs for each parameter set (see supplementary spreadsheet).*

Supplementary Table 12. *Trait heritability observed in the simulations.*

Target h2	ω	Polygenicity	Median h2 (min-max) at the end of step 2	Median h2 (min-max) at the end of step 3
0.25	5	High	0.256 (0.234-0.276)	0.237 (0.204-0.266)
0.25	10	High	0.259 (0.245-0.280)	0.242 (0.223-0.268)
0.5	3	High	0.490 (0.461-0.519)	0.448 (0.419-0.488)
0.5	5	High	0.495 (0.470-0.520)	0.469 (0.414-0.503)
0.5	5	Low	0.478 (0.400-0.559)	0.422 (0.367-0.506)
0.5	10	High	0.508 (0.485-0.548)	0.488 (0.459-0.516)
0.5	10	Low	0.507 (0.458-0.552)	0.471 (0.412-0.52)

Supplementary Table 13. *Correlation between Betas for various ancestry pairs (see supplementary spreadsheet).*

References

1. Baumdicker, F. *et al.* Efficient ancestry and mutation simulation with msprime 1.0. *Genetics* **220**, iyab229 (2022).
2. Nelson, D. *et al.* Accounting for long-range correlations in genome-wide simulations of large cohorts. *PLOS Genet.* **16**, e1008619 (2020).
3. Haller, B. C. & Messer, P. W. SLiM 4: Multispecies Eco-Evolutionary Modeling. *Am. Nat.* **201**, E127–E139 (2023).
4. Gilbert, E. *et al.* The Newfoundland and Labrador mosaic founder population descends from an Irish and British diaspora from 300 years ago. *Commun. Biol.* **6**, 469 (2023).
5. Sanjak, J. S., Sidorenko, J., Robinson, M. R., Thornton, K. R. & Visscher, P. M. Evidence of directional and stabilizing selection in contemporary humans. *Proc. Natl. Acad. Sci.* **115**, 151–156 (2018).
6. Yair, S. & Coop, G. Population differentiation of polygenic score predictions under stabilizing selection. *Philos. Trans. R. Soc. B Biol. Sci.* **377**, 20200416 (2022).
7. Vallini, L. *et al.* Genetics and Material Culture Support Repeated Expansions into Paleolithic Eurasia from a Population Hub Out of Africa. *Genome Biol. Evol.* **14**, evac045 (2022).
8. Marchi, N. *et al.* The genomic origins of the world's first farmers. *Cell* **185**, 1842-1859.e18 (2022).
9. Bortolini, E. *et al.* Early Alpine occupation backdates westward human migration in Late Glacial Europe. *Curr. Biol.* **31**, 2484-2493.e7 (2021).
10. Lazaridis, I. *et al.* Genomic insights into the origin of farming in the ancient Near East. *Nature* **536**, 419–424 (2016).
11. Patterson, N. *et al.* Large-scale migration into Britain during the Middle to Late Bronze Age. *Nature* **601**, 588–594 (2022).
